# Genomic analysis of Hepatitis B virus and its association with disease manifestations in Bangladesh

**DOI:** 10.1371/journal.pone.0218744

**Published:** 2019-06-28

**Authors:** Ruksana Raihan, Sheikh Mohammad Fazle Akbar, Mamun Al Mahtab, Kazuaki Takahashi, Junya Masumoto, Shahina Tabassum, Kok Keng Tee, Rosmawati Binti Mohamed

**Affiliations:** 1 Department of Medicine, Faculty of Medicine, University of Malaya, Kuala Lumpur, Malaysia; 2 Department of Pathology, Ehime University Proteo-Science Center, Ehime University Graduate School of Medicine, Toon City, Ehime, Japan; 3 Department of Hepatology, Bangabandhu Sheikh Mujib Medical University, Dhaka, Bangladesh; 4 Department of Medical Sciences, Tokyo-Shinagawa Hospital, Shinagawa, Tokyo, Japan; 5 Department of Virology, Bangabandhu Sheikh Mujib Medical University, Dhaka, Bangladesh; 6 Department of Medical Microbiology, Faculty of Medicine, University of Malaya, Kuala Lumpur, Malaysia; 7 School of Healthcare and Medical Sciences, Sunway University, Bandar Sunway, Selangor Darul Ehsan, Malaysia; Centre de Recherche en Cancerologie de Lyon, FRANCE

## Abstract

The direct cytopathic effects of the hepatitis B virus (HBV) on subsequent liver damage are not fully understood in HBV-infected patients. However, associations between the prevalence of various HBV genotypes and the extent of liver damage have been reported from different parts of the world. The purpose of this study was to determine the distribution of HBV genotypes in patients with chronic HBV infection in Bangladesh, a country of 160 million people, of which approximately 3–6 million are chronically infected HBV patients. In addition, whole and partial genome sequencing of HBV was performed to evaluate the relationship between HBV mutations and genotypes. We found that 42% of the patients with low HBV DNA and normal levels of alanine aminotransferase (ALT) had HBV genotype D. In contrast, the HBV genotype C was dominant among patients with high HBV DNA levels (>2000 IU/ml) and elevated ALT and in patients with liver cirrhosis (LC) and hepatocellular carcinomas (HCC). Whole and partial genome sequences of HBV revealed that most patients with LC and HCC had HBV genotype C with mutations at the T1762/A1764 positions. It seems that Bangladesh represents a borderline country, situated within East Asia, which mainly consists of individuals with HBV genotypes B and C, whereas in the western parts of Asia, HBV genotypes A and D are prevalent. Bangladesh is, therefore, an excellent model for the comparison of the pathophysiology of three major HBV genotypes in a single population. The findings of this study suggest a possible association between HBV viral factors and the extent of liver damage in chronic HBV-infected patients.

## Introduction

Hepatitis B virus (HBV) is one of the most common viral infections in humans, and is endemic to Asia, the Pacific Islands, Africa, Southern Europe, and Latin America. Approximately 2 billion people worldwide have serological markers for past or present infection with HBV. Of this subset, approximately 248 million people are chronically infected with HBV and express the hepatitis B surface antigen (HBsAg) [1]. Many patients, chronically infected with HBV, may lead uneventful lives without any visible signs of liver damage. However, a considerable number of chronically infected HBV subjects develop hepatic inflammation (chronic hepatitis [CHB]), fibrosis with alterations of the lobular structure of the liver (cirrhosis of the liver [LC]), and/or hepatocellular carcinomas (HCC) resulting in about 1 million HBV-related death each year [2].

Hepatitis B is a noncytopathic virus, and the direct role of the HBV as well as HBV-related antigens has yet to be determined in related liver diseases. However, investigators from different regions of the world have recently discovered associations between HBV genotypes and the extent of liver damage experienced [3–6]. Almost all major genotypes (A to F) were detected in a study on patients in the USA. However, the study merely reflected the country of origin of the patients and did not address any possible association between HBV genotypes and the extent of liver damage [7]. In contrast, studies from Taiwan and Japan have shown that HBV genotype C is associated with a higher risk of liver diseases than genotype B [8, 9]. Studies from Europe have shown that HBV genotype D is related to more severe liver diseases, whereas, HBV genotype A leads to more chronicity [10, 11]. However, little is known about the association between the HBV genotype and the extent of liver damage in developing and resource-constrained countries that harbor with more than 80% of the chronically HBV-infected population.

Bangladesh has a population of 160 million people, of which, about 3–6 million are chronically infected with HBV. Previous studies, although with a small sample size, have shown that three major HBV genotypes exist here; genotypes A, C, and D [12–15]. This provides an opportunity to compare the association between three different genotypes and HBV-induced liver disease, unlike most studies from Asia and Europe, which only compare the two major HBV genotypes [8–11, 16,17]. In addition, mutations at T1762/A1764 are associated with progressive liver disease [18, 19]; however, the association between these mutations and the extent of liver damage in Bangladesh or other developing countries remains unknown.

Thus, in this study, we determined the genotypes of HBV present in our patients and conducted full and partial genome sequencing to explore mutations at specific regions of the genome. These data were analyzed to determine if there existed a relationship between these factors (genotype and mutations) and HBV-induced liver disease in chronically infected HBV individuals in Bangladesh.

## Materials and methods

### Study design

Subjects were selected from a patient pool of 800 chronically infected HBV individuals of Bangladeshi origin. The pool was originally developed to study the pathophysiology of HBV-related liver disease in Bangladesh [20–22] and conduct phase I/II/III clinical trials with a novel therapeutic immune modulatory vaccine for chronic hepatitis B patients [22]. Patients expressing markers for hepatitis C, human immunodeficiency virus (HIV), or acute infections with the hepatitis A or E viruses (assessed by IgM type antibodies to HAV and HEV) were excluded from the study. Patients with concomitant autoimmune diseases and non-alcoholic fatty liver disease were also excluded from the analyses. The patients were from three different hospitals in Dhaka, Bangladesh: (1) Bangabandhu Sheikh Mujib Medical University (BSMMU) (2) Farabi General Hospital, and (3) Lab-Aid Specialized Hospital. The patients in this cohort were consulting physicians for the following reasons: (1) HBsAg positivity on routine health check-up (2) development of clinical and biochemical features of chronic hepatitis and subsequent advanced management, and/or (3) emergence of complications like LC and HCC. Of these 800 patients, final analyses were conducted on 360 patients with complete clinical data and on which assessments of HBV genotypes could be accomplished.

### Grouping of patients

The chronically infected HBV patients were initially classified as those without or with LC and HCC (Group 3). Those without LC and HCC were further divided into two groups based on their levels of HBV DNA and alanine aminotransferase (ALT). Group 1 consisted of patients with low HBV DNA levels (<2000 IU/ml) and ALT persistently below the upper limit of normal (ULN). Group 2 consisted of patients with high HBV DNA levels (>2000 IU/ml) and ALT levels above ULN (Table 1) (S1–S3 Tables)

**Table 1:** Grouping of patients with chronic HBV infection.

Parameters	Group 1 (n = 48) (HBV DNA<2000 IU/ml, ALT<ULN)	Group 2 (n = 74) (HBV DNA>2000 IU/ml, ALT>ULN)	Group 3 (n = 73) Liver Cirrhosis and Hepatocellular Carcinoma
**Age**	28.1 ± 8.5	27.4 ± 9.5	44.3 ± 14.58
**Male: Female**	38:10	66:8	68:5
**HBV DNA (IU/ml)**	802 ± 623	2.2×10^10^ ± 1.1×10^10^	1.6×10^10^ ± 1.1 x 10^12^
**ALT (IU/L)**	30 ± 7 (16–40)	65 ± 39 (43–340)	81 ± 61 (23–315)
**HBeAg/Anti-HBe**	4:44	27:47	15:58

ULN: Upper limit of normal; ALT: Alanine aminotransferase; ULN of ALT: 42 IU/L; The values are shown as mean ± standard deviation, except HBV DNA which has been shown as median with range

### Ethical permission

The study was approved by the ethical review board of Bangabandhu Sheikh Mujib Medical University (BSMMU) and the ethical Committee of the Farabi General and Lab-Aid Specialized Hospitals. All patients gave their informed verbal consent for the study after getting a full explanation of the nature and purpose of the study. In case of minors, the consent was taken from their legal guardians.

### HBV serology

Hepatitis B surface (HBsAg), hepatitis B e antigen (HBeAg), and antibody to hepatitis B core antigen (anti-HBc), antibody to HBeAg (anti-HBe), IgM type antibody to HBc (IgM anti-HBc), antibody to hepatitis C virus (anti-HCV), and IgM type antibody to hepatitis A virus (IgM anti-HAV), and E viruses (IgM anti-HEV) were measured using commercial kits (DRG International INC, Springfield, NJ, USA) according to the manufacturer’s instructions [20–22].

### Partial sequencing

To determine the HBV genotype, HBV DNA was extracted from 100 μL samples of stored blood serum using SMITEST EX-R&D (Genome Science Laboratories, Fukushima, Japan). Then, a nested PCR was performed using the Prime STAR1 GXL polymerase (Takara Bio Inc., Shiga, Japan) as previously described [23] and oligonucleotide primers listed in Table 2. The obtained PCR product (fragment A) was directly sequenced using specific primers to determine the nucleotide sequence at the nt715-1482 and nt1600-1790 sites. Samples were sequenced using the Big-Dye Terminator v3.1 Cycle Sequencing Kit (Thermo Fisher Scientific K.K., Kanagawa, Japan) and defined using an auto-sequencer and Applied Biosystems 3730xl DNA Analyzer (Thermo Fisher Scientific K.K., Kanagawa, Japan).

**Table 2:** The primers used to determine HBV genotypes and for full genome of sequencing of HBV.

**Determination of HBV Genotypes**				
1st-Sense		X1-1a: TGCCAAGTGTTTGCTGACGC (1174–1193)		
		X1-1b: TGCTGAYGCAACCCCCACTG (1185–1204)		
		X1-1c: GCTCCTCTGCCGATCCATAC (1252–1271)		
1st-Anti-Sense		X1-2: AAAGTTGCATGGTGCTGGTG (1804–1823)		
		X1-2b: GAGATGATTAGGCAGAGGTG (1826–1845)		
2nd-Sense		X2-1a: TCCTCTGCCGATCCATACTGC (1254–1274)		
		X2-1b: ACACMTCCTTTCCATGGCTGC (1361–1381)		
		X2-1c: ATACATCRTTTCCATGGCTGC (1361–1381)		
2nd-Anti-sense		X27: CAGACCAATTTATGCCTACAGC (1780–1801)		
**Primers for Full Genome of Sequencing of HBV**				
Fragment A	WA-L	1862–1885	Sense	ACTGTTCAAGGGTCCAAGCTGTGC
	WA-R	1806–1829	Antisense	AGCAAAAAGTTGCATGGTGCTGGT
	FA2R	217–240	Antisense	GGTATTGTGAGGADDYTTGTCAAC
	FA4L	801–820	Sense	GTATTGGGGGCCAAGTCTGT
	FA4LAS	801–820	Antisense	ACAGACTTGGCCCCCAATAC
	FA3L	107–124	Sense	CTGCTGGTGGCTCCAGTT
	WA-2R	1781–1802	Antisense	CAGACCAATTTATGCCTACACG
	WA-2L	1887–1908	Sense	GGTGGCTTTRGGRCATGGACAT
	PAAS	455–474	Antisense	CAAGGTTATGTTGCCGTTTG
	B260	260–279	Antisense	AGAAATTGAGAGAAGTCCA
	B1260	1260–1279	Sense	GCCGATCCATACTGGGAAC
	B2466	2466–2485	Antisense	GTAAAGTTTCCCACCTTATG
	B2830	2830–2849	Antisense	ATGCTGTAGCTCTTGTTCCC
Fragment B	S1	1414–1434	Sense	ACGTCCTTTGTTTACGTCCCG
	S2	1436–1456	Sense	CGGCGCTGAATCCCGCGGACG
	S3	1489–1508	Sense	CGGCTTCTCCGTCTGCCGTA
	S4	1527–1547	Sense	CACCTCTCTTACGCGGACTC
	AS1	2130–2110	Antisense	TCAAATTACTAATTCCCTGGAT
	AS2	2160–2140	Antisense	CTGACTACTAATTCCCTGGAT
	AS3	2185–2165	Antisense	TAGGCCCATATTAACATTGAC
	AS4	2098–2078	Antisense	CATCAACTCACCCCAACACAG

### HBV full-length genome sequencing

To determine the full-length genome sequence of the HBV DNA, direct sequencing of the partially sequenced fragment A using the primers WA-2L, FA3L, P4AS, B260, B2466, B2830, FA2R, and FA4LAS was conducted. To further sequence the portion missing from the circular HBV DNA (fragment B), a nested PCR was performed on the extracted DNA using Prime STAR1 GXL polymerase, the primer set S1, S2, AS2, and AS2, and the inner primers S2, S3, AS2, and AS3. Fragment B was directly sequenced using primers S4 and AS4 in both directions. For credible homology analysis, full-length genome sequences were used [23, 24]. The primers used are listed in Table 2.

### Genetic analysis and phylogenetic analysis

Genetyx-Mac version 17 (software, Genetyxs Co., Tokyo, Japan) was used for sequence alignment, mutation, mapping, homology, and phylogenetic analyses of the obtained nucleotide sequences. A bootstrap NJ method was used with 1000 re-samplings to produce a phylogenetic tree. GenBank accession numbers for all newly sequenced genomes will be submitted (23, 24).

## Statistical analysis

The statistical analysis evaluated the proportions of patients with different genotypes and the association to disease progression as well as the proportion of mutants according to the disease progression and the association of the genotype C to the presence of mutations in HBV genome. These frequency analyses were conducted using the program PRISM version 5, using the Fisher Exact test, and also Chi-Square test when the cell number was above 4.

## Results

### Prevalence of three major HBV genotypes in Bangladeshi chronic HBV-infected patients

Of the 360 patients analyzed, HBV genotype A, C, and D were found in 65 (18%), 153 (43%), and 142 (39%) patients, respectively. The data indicated that there was a high prevalence of all three HBV genotypes within the chronically infected HBV population of Bangladesh.

### Association of HBV genotype with extents of liver damages

Of the 360 patients analyzed, 195 patients could be grouped into one of these three groups (Group-1: 48 patients with low HBV DNA and ALT persistently within ULN; Group-2: 74 patients with high HBV DNA and ALT levels above ULN, and Group-3: 73 patients with LC and HCC) (Table 1). The remaining 165 patients could not be included in any of these groups because they either expressed high HBV DNA levels (>2000 IU/ml) with normal ALT (<42IU/L) or low HBV DNA levels (<2000IU/ml) with at least one spike of high ALT (>42IU/L).

The HBV genotype showed marked variability in terms of HBV DNA and ALT levels, and LC or HCC occurrence. Patients in Group 1 mostly correspond to HBV genotypes D (41.7%) and A (33%) (Table 3). However, HBV genotype A was detected in less than 10% of the patients in Groups 2 and 3 (9.4% and 8.2%, respectively); the HBV genotype C was the main genotype found in these two groups. The prevalence of genotype C was significantly higher in Group 2 (60.8%; p = 7.37x10^-5^) and Group 3 (71.2%; p = 4.80x10^-7^) compared to Group 1 (25%), resulting in highly significant differences in both comparisons (p<0.001) (Table 3). Similarly, highly significant differences in proportion of genotype A patients were found in Group 1 compared to Group 2 (p = 9.91x10^-4^) and to Group 3 (p = 4.79x10^-4^).

**Table 3:** HBV genotype distribution among three groups of chronically infected HBV patients.

Diagnosis	Genotype A	Genotype C	Genotype D
Group 1 (n = 48)	16 (33.3%)*	12 (25%)	20 (41.7%)
(HBV DNA<2000 IU/ml, ALT<ULN)			
Group 2 (n = 74)	7 (9.4%)	45 (60.8%)**	22 (29.7%)
(HBV DNA>2000 IU/ml, ALT>ULN)			
Group 3 (n = 73)	6 (8.2%)	52 (71.2%)**	15 (20.5%)
Liver Cirrhosis and Hepatocellular Carcinoma			

*p<0.001, compared to Group-2 and Group-3 (Fisher Exact Test)

**p<0.001, compared to Group-1 (Fisher Exact Test)

### Association of HBV genome sequences with neighboring countries

**Fig 1:**
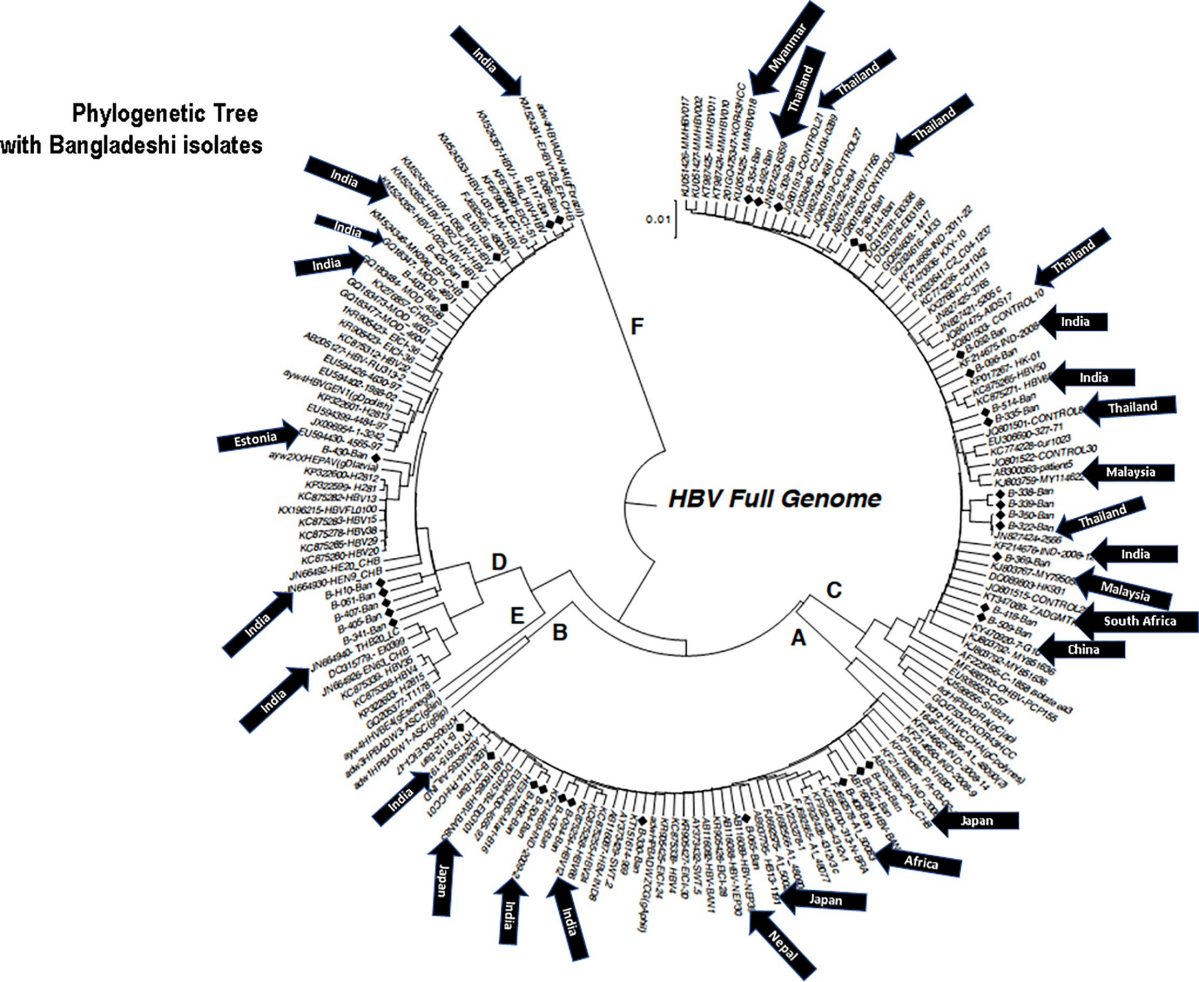
Phylogenetic tree constructed with 38 full genome sequences of hepatitis B virus from Bangladesh. The sequences of Bangladeshi isolates are shown in the phylogenetic tree indicated by black diamond markings. The countries that have close association with Bangladeshi HBV isolates are also indicated with an arrow and the name of the country.

Full genome sequences of HBV were performed in 38 patients with chronic HBV infection (11, 11, and 16 patients with HBV genotypes A, D, and C), respectively. The phylogenetic tree was constructed with the data from these 38 isolates. As shown in Fig 1, the Bangladeshi HBV isolates showed close sequence homology with HBV strains from India, Myanmar, Nepal, and Thailand, the neighboring countries of Bangladesh. Furthermore, some HBV isolates exhibited close homology with strains of Malaysia, China, and Japan, the Asian countries around Bangladesh and countries with a considerable migrant population from Bangladesh. Interestingly, some HBV isolates also showed considerable homology with HBV isolates of South Africa and Estonia.

### High prevalence of T1762/A1764 mutations in patients with progressive liver disease

**Fig 2:**
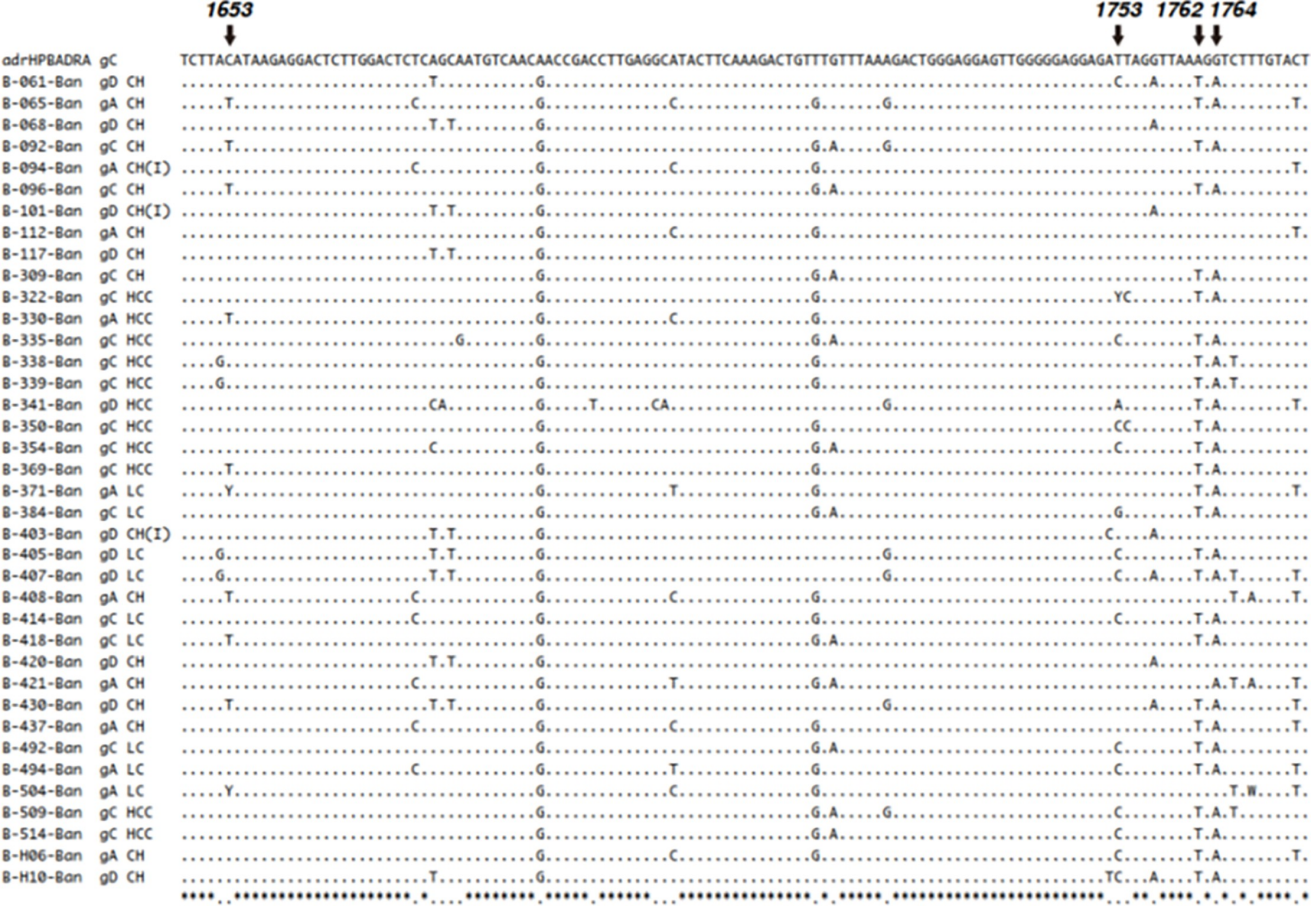
Mutations at 1762 and 1764 of Bangladeshi isolates. Full genome sequences were created in 38 HBV isolates from 38 patients with chronic HBV infection. At the top, the sequence of adrHPBADRA has been shown. B-061-Ban to B-H10-Ban (38 isolates) indicate the identification number of Bangladeshi isolates. The genotypes are shown as gA, gC, and gD on the right side of the identification number. The diagnosis of each isolate has been shown as: Chronic hepatitis (CH) with low HBV DNA (<2000 IU/ml) and normal ALT (Group-1); CH with high HBV DNA (>2000 IU/ml) and elevated ALT (>upper limit of normal) (Group-2); LC, cirrhosis of liver and HCC, hepatocellular carcinoma (Group-3). Also, G1895 mutation was found in 10 patients in our cohort (S1 Fig).

Increased mutations of the HBV genome at nt positions 1762 and 1764 have been reported in patients from Japan, China, and some other countries with advanced liver diseases, such as LC and HCC [18, 19, 24]. However, this issue has never been addressed in Bangladeshi patients with advanced HBV-related liver disease (LC and HCC). One of the main objectives of this study was to elucidate if these mutations were also common in patients with LC and HCC in Bangladesh. Whole genome sequencing was conducted in 38 patients with chronic HBV infection, including 3, 15, and 20 patients of group 1, 2, and 3, respectively. Mutations at T1762/A1764 were not found in any of the group 1 patients (Fig 2). However, these mutations were found in 9 (60%) of the 15 group 2 patients. In addition, the mutations were detected in 18 (90%) of the 20 group 3 patients. Moreover, of the 20 group 3 patients studied, 13 had the HBV genotype C and all of them expressed mutations at T1762/A1764 (Fig 2) indicating a higher prevalence of mutations at T1762/A1764 in patients with progressive liver diseases (Fig 2, Table 4). The presence of mutations was significantly higher in Group 3 patients when compared to the rest of the patients together (Group 1 & 2; p = 0.011).

**Table 4:** Prevalence of T1762/A1764 mutations in various forms of liver disease.

Group	Prevalence of genotype		Mutations at T1762/A1764	Total
Group 1 (n = 3) (HBV DNA<2000 IU/ml, ALT<ULN)	A	1 (33.3%)	0	0%
	D	2 (66.7%)	0	
Group 2 (n = 15) (HBV DNA>2000 IU/ml, ALT>ULN)	A	6 (40%)	3 (20%)	9 (60%)
	C	3 (20%)	3 (20%)	
	D	6 (40%)	3 (20%)	
Group 3 (n = 20) Liver Cirrhosis and Hepatocellular Carcinoma	A	4 (20%)	2 (10%)	18 (90%)
	C	13 (65%)	13 (65%)	
	D	3 (15%)	3 (15%)	
Total N = 38				27 (71.05%)

### Partial HBV genomic sequencing supported prevalence of T1762/A1764 mutations in HBV genotype C

The full genome sequencing was accomplished in only 38 patients with chronic HBV infection. However, this provided insight into the association between HBV genotype C and mutations at T1762/A1764 in patients with progressive liver diseases. We further searched for mutations at T1762/A1764 in 73 additional patients (6, 36, and 31 patients of group 1, 2, and 3, respectively) with different levels of liver injury and the HBV genotype C, to maximize the scientific significance of our observation. Mutations at T1762/A1764 were detected in 16% and 22.2% of group 1 and 2 patients, respectively. Interestingly, mutations at T1762/A1764 were detected in 29 of 31 (93.5%) of the group 3 patients, suggesting a possible role of mutant HBV on HBV genotype C in patients with progressive liver diseases (Table 5). When uniquely genotype C patients were taken into account, a higher proportion of mutations, reaching a highly significant statistical difference (p<0.001), was found among patients with disease progression to LC and HCC (Group 3).

**Table 5:** Association between HBV genotype C and increased prevalence of mutations at T1762/A1764.

Group	Mutations at T1762/A1764
Group 1 (n = 6) (HBV DNA<2000 IU/ml, ALT<ULN)	1 (16%)
Group 2 (n = 36) (HBV DNA>2000 IU/ml, ALT>ULN)	8 (22.2%)
Group 3 (n = 31) Liver Cirrhosis and Hepatocellular Carcinoma	29 (93.5%)

## Discussion

Like most developing and resource-constrained countries, there is a lack of nation-wide surveys on HBV prevalence and mortality in Bangladesh. However, epidemiological data and published literature indicate that there are approximately 3–6 million chronically infected HBV individuals in Bangladesh. With recent economic developments and the impact of social media, the attention of the masses has been directed towards HBV. In addition, Bangladesh is a signatory to eliminate HBV by 2030 as proposed by the World Health Organization. However, there is a lack of information on HBV virology and HBV-induced liver damage in Bangladesh and thus, a proper management strategy and surveillance system are yet to be implemented.

Hepatitis B Virus genotypes in Bangladesh have been described by several investigators. In 2008, Raihan et al. [12] reported the prevalence of HBV genotypes C and D in Bangladesh. Similarly, [13] found a high prevalence of HBV genotype D (73.7%) and low prevalence of HBV genotype C (10.5%) in 19 Bangladeshi patients. In contrast, two proceeding studies on 39 and 53 patients, respectively, found that the HBV genotype C was the primary genotype in Bangladeshi individuals [14, 15]. However, the association between HBV genotype and HBV-related liver diseases in Bangladeshi patients remained unknown.

Thus, in this study, all the HBV infected patients were selected via strict criteria to maximize the clinical significance of our study. We found that Bangladeshi patients have three major HBV genotypes (A, C and D) in considerable proportions. Migration from Mongolia (Mongol invasion in 16th century) and occupation of Bangladesh by the East-India Company (18th century) may be related to the prevalence of multiple HBV genotypes of West Asia, Far-East Asia, and Europe in Bangladesh. Thus, Bangladesh may be an effective model for determining the pathophysiology of the three major HBV genotypes.

We elucidated the association between HBV genotypes and HBV-related liver diseases. Most of patients with high HBV DNA levels and elevated ALT (60.8%), and LC and HCC (71.2%) had HBV genotype C, whereas, HBV genotype D and A were mostly prevalent in patients with persistently low HBV DNA and normal ALT (75%) levels. Although an association between genotype C and more severe forms of HBV-related liver diseases has been shown, the underlying factors and mechanisms of such an association remain unknown and poorly understood. As mentioned by Sumi et. Al. [25], this may reflect the duration of HBeAg-positivity and extents of viral replication. They found that patients with HBV genotype B experienced earlier HBeAg seroconversion, slower progression of liver fibrosis and slowed development of HCC compared to patients with HBV genotype C, however, the life long risk of progression to advanced fibrosis and development of HCC was not significantly different among HBV genotype B and C [25]. As we found 3 major HBV genotype, A, C and D among chronic HBV-infected patients in Bangladesh, more insight would emerge regarding this in future.

We approached this question from two perspectives: virologically and immunologically. Pre-core/core promoter mutants at positions T1762/A1764 of the HBV genome are associated with severe forms of liver diseases as well as hepatocarcinogenesis [18, 19, 24]. Thus, these mutations were searched for via the full genome sequencing of HBV in 38 patients with chronic HBV infection. The prevalence of T1762/A1764 mutations were detected in significantly higher frequencies in patients with high HBV DNA and ALT levels, and LC and HCC patients than those with low HBV DNA and normal ALT levels (p<0.05) (Fig 2). Further analyses by partial sequencing revealed that most of the patients with mutations at T1762/A1764 had HBV genotype C. This was further confirmed by partial sequencing of the HBV genome to search for these mutations in 31 patients with LC, HCC, and HBV genotype C. Twenty-nine of these patients expressed mutations at T1762/A1764. Thus, there is a close association between HBV genotype C with mutations at T1762/A1764 and progressive liver diseases in Bangladeshi HBV-infected patients.

As HBV is a non-cytopathic virus, there is a need to clarify how HBV genotype C with mutations at T1762/A1764 encourage the progression of severe liver diseases. An immunological study is now in progress in our laboratory to determine the variability in host immunity of different HBV genotypes with and without mutations. The preliminary data indicates increased proinflammatory cytokines and nitric oxide production by various immunocytes in patients with HBV genotype C having mutations at T1762/A1764 (data not shown, pers. comm. Sheikh Mohammad Fazle Akbar).

Nevertheless, this study comes with some limitations. Although, it includes the highest sample size of any Bangladeshi genotype study on HBV [12–15], a national survey of HBV genotypes with a larger sample size is essential. In addition, more patients with inactive HBV in the carrier state should have been included in the study. This is a difficult task as the majority of inactive, chronically infected HBV patients are unaware of their infection; this is the case in almost all developing countries. It may also be necessary to determine what mutations occur in other regions of HBV by enrolling more patients for full genome sequencing. Moreover, future studies need to assess how many group 2 patients develop LC and HCC. To address this issue, we have developed a patient pool system at Bangladesh so that follow-ups can be conducted on the patients in the long-term.

Thus, this study presented indicates that there may be an inherent association between HBV genotype C and mutations at specific sites on the HBV genome in Bangladeshi patient at risk of progression to LC and HCC. More insight on this association may assist in denoting population groups at risk of progressive chronic HBV infections and implementing management strategies.

## Supporting information

S1 File Ethical clearance. (JPG)

S1 Fig G1895 mutation in patients with chronic hepatitis B. (TIF)

S1 Table Profile of patients of Group-1. (DOCX)

S2 Table Profile of patients of Group-2. (DOCX)

S3 Table Profile of patients of Group-3. (DOCX)
