# Supplementary figures and images for: Genomic analysis of Hepatitis B virus and its association with disease manifestations in Bangladesh
Source: PLoS One. 2019 Jun 28;14(6):e0218744. doi: 10.1371/journal.pone.0218744 (PMC6599139; doi:10.1371/journal.pone.0218744)

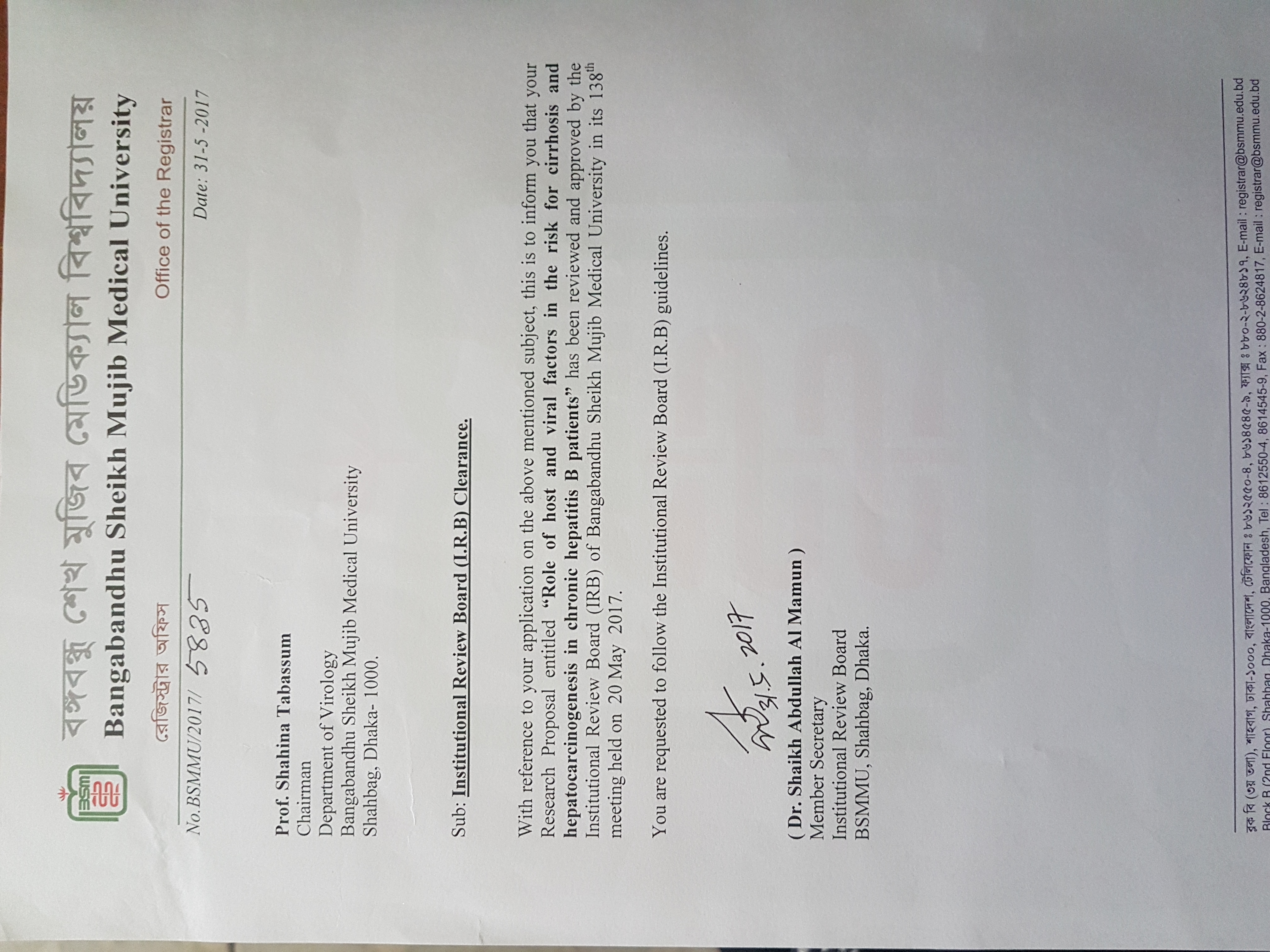

Supplement: S1 File — (JPG) [file pone.0218744.s001.jpg]

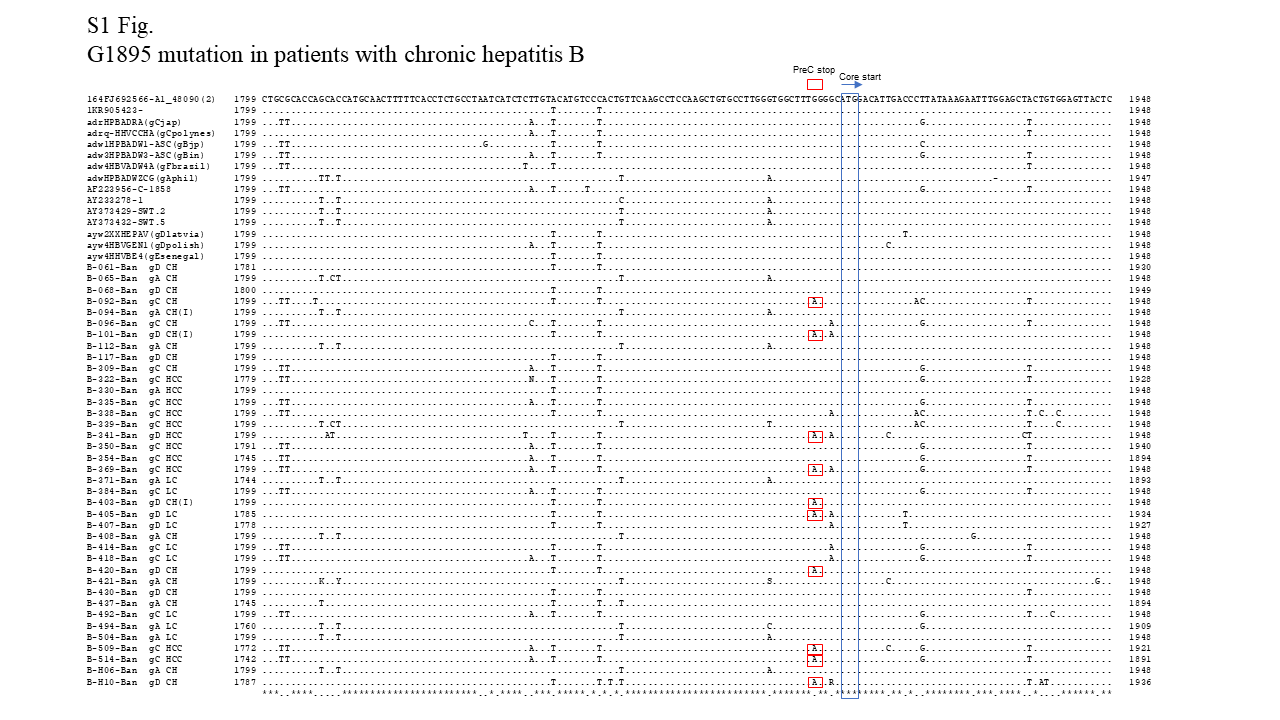

Supplement: S1 Fig — (TIF) [file pone.0218744.s002.tif]
