# Supplementary material for: Genomic analysis of Hepatitis B virus and its association with disease manifestations in Bangladesh
Source: PLoS One. 2019 Jun 28;14(6):e0218744. doi: 10.1371/journal.pone.0218744 (PMC6599139; doi:10.1371/journal.pone.0218744)
Supplement: S1 Table — (DOCX) [file pone.0218744.s003.docx]

**SI Table**

Profile of patients of Group-1

| **Age**  **(Years)** | **ALT (IU/ml)** | **DNA (IU/ml)** |
| --- | --- | --- |
| 26 | 28 | 2.00E+01 |
| 35 | 22 | 2.40E+01 |
| 28 | 41 | 6.60E+01 |
| 21 | 32 | 9.60E+01 |
| 31 | 42 | 1.00E+02 |
| 18 | 21 | 2.00E+02 |
| 17 | 22 | 2.00E+02 |
| 38 | 27 | 2.00E+02 |
| 24 | 34 | 2.00E+02 |
| 34 | 40 | 2.00E+02 |
| 22 | 39 | 2.40E+02 |
| 25 | 25 | 2.80E+02 |
| 22 | 28 | 2.80E+02 |
| 45 | 24 | 3.00E+02 |
| 56 | 32 | 3.40E+02 |
| 36 | 16 | 4.20E+02 |
| 32 | 33 | 4.20E+02 |
| 31 | 34 | 4.20E+02 |
| 35 | 23 | 4.40E+02 |
| 37 | 23 | 4.40E+02 |
| 27 | 19 | 5.40E+02 |
| 20 | 27 | 5.40E+02 |
| 35 | 39 | 5.40E+02 |
| **3** | **40** | **6.00E+02** ***** |
| 25 | 25 | 6.20E+02 |
| 24 | 27 | 6.20E+02 |
| 22 | 41 | 6.40E+02 |
| 25 | 34 | 6.60E+02 |
| 34 | 42 | 6.80E+02 |
| 25 | 20 | 8.20E+02 |
| 48 | 22 | 9.60E+02 |
| 30 | 40 | 8.60E+02 |
| 28 | 42 | 1.08E+03 |
| 20 | 29 | 1.24E+03 |
| 25 | 28 | 1.26E+03 |
| 24 | 26 | 1.34E+03 |
| 27 | 30 | 1.38E+03 |
| 23 | 31 | 1.46E+03 |
| 35 | 33 | 1.56E+03 |
| 22 | 30 | 1.56E+03 |
| 20 | 29 | 1.64E+03 |
| 30 | 16 | 1.68E+03 |
| 24 | 30 | 1.74E+03 |
| 25 | 35 | 1.82E+03 |
| 28 | 34 | 1.88E+03 |
| 28 | 24 | 1.88E+03 |
| 28 | 37 | 2.00E+03 |
| 32 | 39 | 2.00E+03 |

*****The median value of HBV DNA has been shown by red color
