# Supplementary material for: Genomic analysis of Hepatitis B virus and its association with disease manifestations in Bangladesh
Source: PLoS One. 2019 Jun 28;14(6):e0218744. doi: 10.1371/journal.pone.0218744 (PMC6599139; doi:10.1371/journal.pone.0218744)
Supplement: S2 Table — (DOCX) [file pone.0218744.s004.docx]

**S2 Table**

Profile of patients of Group-2

| **Age**  **(Years)** | **ALT (IU/ml)** | **DNA (IU/ml)** |
| --- | --- | --- |
| 24 | 45 | 2.10E+03 |
| 32 | 51 | 2.20E+03 |
| 22 | 45 | 2.40E+03 |
| 24 | 45 | 2.40E+03 |
| 33 | 72 | 2.40E+03 |
| 28 | 44 | 2.60E+03 |
| 22 | 56 | 2.80E+03 |
| 25 | 43 | 2.94E+03 |
| 17 | 45 | 3.60E+03 |
| 42 | 62 | 3.60E+03 |
| 43 | 43 | 3.80E+03 |
| 42 | 69 | 3.80E+03 |
| 19 | 61 | 4.00E+03 |
| 28 | 49 | 4.80E+03 |
| 35 | 43 | 5.20E+03 |
| 40 | 60 | 5.60E+03 |
| 28 | 62 | 6.60E+03 |
| 42 | 48 | 7.20E+03 |
| 32 | 51 | 8.40E+03 |
| 29 | 54 | 1.06E+04 |
| 21 | 340 | 1.22E+04 |
| 22 | 47 | 1.24E+04 |
| 24 | 101 | 1.40E+04 |
| 21 | 51 | 1.50E+04 |
| 30 | 46 | 2.00E+04 |
| 30 | 53 | 2.20E+04 |
| 39 | 55 | 2.20E+04 |
| 41 | 53 | 3.40E+04 |
| 22 | 56 | 4.00E+04 |
| 23 | 56 | 4.00E+04 |
| 40 | 58 | 4.00E+04 |
| 26 | 57 | 4.40E+04 |
| 32 | 104 | 4.80E+04 |
| 35 | 60 | 6.00E+04 |
| 38 | 60 | 6.60E+04 |
| 26 | 65 | 1.04E+05 |
| **31** | **51** | **1.12E+05*** |
| 25 | 77 | 1.16E+05 |
| 19 | 122 | 2.20E+05 |
| 40 | 65 | 2.60E+05 |
| 19 | 52 | 3.00E+05 |
| 21 | 54 | 3.20E+05 |
| 11 | 47 | 4.00E+05 |
| 37 | 64 | 5.20E+05 |
| 58 | 53 | 1.86E+06 |
| 27 | 57 | 1.98E+06 |
| 27 | 44 | 2.00E+06 |
| 34 | 48 | 3.72E+06 |
| 30 | 105 | 6.40E+06 |
| 30 | 100 | 6.40E+06 |
| 23 | 45 | 8.40E+06 |
| 45 | 44 | 1.28E+07 |
| 26 | 67 | 1.86E+07 |
| 21 | 56 | 3.60E+07 |
| 22 | 60 | 5.60E+07 |
| 29 | 70 | 1.84E+08 |
| 20 | 74 | 2.00E+08 |
| 24 | 83 | 2.00E+08 |
| 35 | 79 | 5.60E+08 |
| 17 | 74 | 2.00E+09 |
| 22 | 44 | 3.00E+09 |
| 18 | 55 | 3.80E+09 |
| 31 | 61 | 5.20E+09 |
| 18 | 49 | 6.00E+09 |
| 23 | 130 | 8.20E+09 |
| 10 | 47 | 1.52E+10 |
| 18 | 44 | 3.60E+10 |
| 7 | 54 | 5.20E+10 |
| 39 | 170 | 8.60E+10 |
| 28 | 46 | 1.36E+11 |
| 24 | 56 | 2.20E+11 |
| 28 | 58 | 2.20E+11 |
| 9 | 46 | 2.80E+11 |
| 7 | 49 | 5.60E+11 |

*****The median value of HBV DNA has been shown by red color
