# Supplementary material for: Genomic analysis of Hepatitis B virus and its association with disease manifestations in Bangladesh
Source: PLoS One. 2019 Jun 28;14(6):e0218744. doi: 10.1371/journal.pone.0218744 (PMC6599139; doi:10.1371/journal.pone.0218744)
Supplement: S3 Table — (DOCX) [file pone.0218744.s005.docx]

**S3 Table**

Profile of patients of Group-3

| **Age** | **ALT (IU/ml)** | **DNA (IU/ml)** |
| --- | --- | --- |
| 35 | 44 | 1.60E+03 |
| 40 | 107 | 2.30E+03 |
| 50 | 72 | 3.00E+03 |
| 16 | 36 | 4.50E+03 |
| 65 | 23 | 7.00E+03 |
| 57 | 65 | 1.05E+04 |
| 57 | 54 | 1.05E+04 |
| 50 | 27 | 1.40E+04 |
| 38 | 71 | 3.35E+04 |
| 40 | 54 | 4.45E+04 |
| 17 | 47 | 5.10E+04 |
| 17 | 112 | 5.10E+04 |
| 55 | 38 | 6.60E+04 |
| 46 | 67 | 6.70E+04 |
| 35 | 36 | 7.10E+04 |
| 50 | 128 | 5.10E+05 |
| 48 | 200 | 7.80E+05 |
| 20 | 90 | 8.20E+05 |
| 30 | 23 | 1.05E+06 |
| 70 | 83 | 1.93E+06 |
| 65 | 150 | 2.70E+06 |
| 50 | 85 | 3.30E+06 |
| 45 | 42 | 3.33E+06 |
| 35 | 56 | 3.60E+06 |
| 50 | 68 | 5.20E+06 |
| 35 | 167 | 7.50E+06 |
| 42 | 77 | 8.20E+06 |
| 55 | 56 | 9.60E+06 |
| 24 | 67 | 1.00E+07 |
| 30 | 36 | 1.04E+07 |
| 51 | 156 | 1.08E+07 |
| 50 | 32 | 1.20E+07 |
| 56 | 76 | 1.20E+07 |
| 59 | 87 | 1.24E+07 |
| 54 | 62 | 1.40E+07 |
| 54 | 34 | 1.40E+07 |
| **52** | **43** | **1.42E+07*** |
| 52 | 25 | 1.69E+07 |
| 55 | 72 | 1.82E+07 |
| 30 | 56 | 3.56E+07 |
| 34 | 45 | 5.00E+07 |
| 40 | 38 | 5.20E+07 |
| 60 | 82 | 6.00E+07 |
| 55 | 39 | 6.89E+07 |
| 55 | 98 | 7.00E+07 |
| 32 | 65 | 7.11E+07 |
| 20 | 70 | 7.11E+07 |
| 22 | 232 | 7.50E+07 |
| 40 | 158 | 7.56E+07 |
| 45 | 271 | 7.56E+07 |
| 22 | 70 | 8.00E+07 |
| 42 | 315 | 9.56E+07 |
| 33 | 23 | 9.56E+07 |
| 47 | 45 | 9.56E+07 |
| 52 | 29 | 9.56E+07 |
| 40 | 80 | 1.00E+08 |
| 17 | 27 | 1.00E+08 |
| 35 | 34 | 1.07E+08 |
| 32 | 78 | 1.20E+08 |
| 65 | 37 | 1.44E+08 |
| 32 | 83 | 1.62E+08 |
| 60 | 247 | 2.66E+08 |
| 60 | 76 | 7.11E+08 |
| 20 | 128 | 7.11E+08 |
| 33 | 32 | 7.11E+08 |
| 56 | 227 | 9.11E+08 |
| 56 | 82 | 1.04E+09 |
| 55 | 56 | 1.20E+09 |
| 37 | 43 | 1.69E+09 |
| 60 | 67 | 1.69E+09 |
| 75 | 91 | 5.11E+09 |
| 60 | 58 | 9.56E+09 |
| 65 | 65 | 9.11E+10 |

*****The median value of HBV DNA has been shown by red color
